# DNA methylation analysis of benign and atypical meningiomas: correlation between *RUNX3* methylation and WHO grade

**DOI:** 10.1007/s00432-015-1930-5

**Published:** 2015-02-04

**Authors:** Aleksandra Majchrzak-Celińska, Jarosław Paluszczak, Marlena Szalata, Anna-Maria Barciszewska, Stanisław Nowak, Wanda Baer-Dubowska

**Affiliations:** 1 Department of Pharmaceutical Biochemistry, Poznan University of Medical Sciences, Poznań, Poland; 2 Department of Biochemistry and Biotechnology, Poznan University of Life Sciences, Poznań, Poland; 3 Department and Clinic of Neurosurgery and Neurotraumatology, Poznan University of Medical Sciences, Poznań, Poland

**Keywords:** DNA methylation, Meningioma, *RUNX3*, *NDRG2*, MSP, PSQ

## Abstract

**Purpose:**

Although meningiomas are common central nervous system tumors, the biomarkers allowing early diagnosis and progression are still needed. The aim of this study was to evaluate the methylation status of 12 cancer-related genes, namely *ERCC1*, *hMLH1*, *ATM*, *CDKN2B* (*p15INK4B*), *p14ARF*, *CDKN2A* (*p16INK4A*), *RASSF1A*, *RUNX3*, *GATA6*, *NDRG2*, *PTEN*, and *RARβ*, in 44 meningioma samples of WHO grade I and II.

**Methods:**

All genes were analyzed using methylation-specific polymerase chain reaction, while pyrosequencing (PSQ) was used to study *NDRG2* promoter methylation.

**Results:**

The most frequently methylated genes in both types of meningiomas were *p14ARF*, *RASSF1A,* and *p15INK4B*. *RUNX3*, *GATA6*, and *p16INK4A* were methylated to a lesser extent, whereas *ATM* and *RARβ* were found to be methylated in a marginal number of patients. The *ERCC1*, *hMLH1*, *NDRG2*, and *PTEN* genes were unmethylated in all cases. Although tumors of the same grade according to WHO criteria had different genes methylated, the number of methylated genes for each individual patient was low. *RUNX3* methylation significantly correlated with meningioma WHO grade, therefore, can be considered as a potential indicator of tumor aggressiveness. The sequence of *NDRG2* chosen for PSQ analysis was found methylated in the majority of meningiomas; however, the methylation level was only slightly elevated as compared to non-cancerous brain.

**Conclusions:**

Overall, the results of this study confirm that DNA methylation plays an important role in the pathogenesis of meningiomas. Further investigations, particularly concerning *RUNX3* methylation, are necessary in order to assess the clinical usefulness of the methylation analysis of the studied genes.

## Introduction

The most common tumor site in the central nervous system is the meninges (35 %) and meningiomas, with the incidence rate of 7.22 per 100,000 persons, are the most common primary brain tumors (Dolecek et al. 2012). Incidence rate for tumors of the meninges is 2.2 times greater in females as compared to males, and the peak incidence occurs in the sixth and seventh decade of life (Dolecek et al. 2012; Lamszus 2004). They are classified according to the World Health Organization (WHO) grading system into grade I, II, or III tumors, and around 80 % of meningiomas are grade I tumors (benign), 10–15 % are grade II (atypical), and 2–5 % are grade III (anaplastic/malignant) (Cahill and Claus 2011). Grade I tumors are mostly curable by surgical resection and have good prognosis; however, some populations of meningiomas with benign histological profiles show malignant behavior. The reason for this inconsistency is yet to be elucidated, and the search for novel diagnostic, prognostic, and predictive biomarkers which could help in meningioma patients stratification and treatment is therefore needed. In addition to alterations in the DNA sequence, epigenetic modifications are closely linked to cancer initiation and progression. Aberrant DNA methylation is one of the major types of epigenetic modifications in cancer (Paluszczak and Baer-Dubowska 2006). Changes in DNA methylation appearing during early stages of malignancy are potentially reversible and in some cases correlate well with tumor characteristics. For instance, *TIMP3* inactivation as a result of gene promoter methylation appears to be involved in meningioma progression as it is associated with more aggressive, high-grade meningioma phenotype (Barski et al. 2010). Recently, three genome-wide methylation analyses of benign, atypical, and malignant meningiomas were performed (Kishida et al. 2012; Vengoechea et al. 2013; Gao et al. 2013). All concluded that aberrant hypermethylation of subset of genes occurs in the early stage of meningiomas and highlighted the possibility of malignancy evaluation by assessing methylation status. However, a crucial methylation marker to predict an aggressive subtype in the early stage of meningiomas remains to be established, as existing data are still limited and often inconsistent. Our previous study has shown that a panel of 12 genes including *ERCC1*, *hMLH1*, *ATM*, *CDKN2B* (*p15INK4B*), *p14ARF*, *CDKN2A* (*p16INK4A*), *RASSF1A*, *RUNX3*, *GATA6*, *NDRG2*, *PTEN*, and *RARβ* might be useful in evaluation of glioma aggressiveness. In this regard, methylation index (MI) of these genes in individual patient, as well as *RUNX3* methylation correlated with glioma WHO grade and higher MI, or aberrant methylation of *RUNX3* indicated more aggressive tumors (Majchrzak-Celińska et al. 2015).

The aim of the present study was to evaluate the possible involvement of the hypermethylation of these genes in the malignant profile of benign and atypical meningiomas.

## Materials and methods

### Patients

The study included 44 patients who were referred to the Department and Clinic of Neurosurgery and Neurotraumatology, Poznan University of Medical Sciences, Poznań, Poland, between January 2010 and September 2013. Patients with a histologically confirmed diagnosis of meningioma at different grades of malignancy (I, I/II or II) and a treatment protocol that included surgical resection were enrolled in the study. Histological types of the tumors as well as tumor grades (according to the 2007 WHO classification criteria) were analyzed in the Laboratory of Neuropathology. Thirty-three patients were diagnosed with benign WHO grade I meningioma, two with grade I/II, and nine with atypical meningiomas (grade II). Women comprised 72.73 % (32/44) of all patients, and the average patients’ age was 56 years, ranging from 31–87. After resection, tumor samples were directly frozen and stored at −80 °C.

Control non-cancerous brain sample originated from a patient with brain hematoma, and blood sample for the analysis of white blood cells methylation was taken from healthy donor. All patients gave informed consent for the analyses to be undertaken, and the study protocol was approved by the Poznan University of Medical Sciences Clinical Research Ethics Committee (approval no. 505/12).

### Isolation of DNA from tumor tissue

DNA was isolated using GeneMATRIX Tissue DNA Purification Kit [EurX, Gdańsk, Poland]. Briefly, 25 mg of tumor tissue was incubated with Lyse T Buffer, then RNase A and proteinase K, which was followed by incubation in 56 °C until the tissue was completely digested. Buffer Sol T and ethanol were then added to provide selective conditions for DNA binding during brief centrifugation, and then, DNA bound to minicolumn was washed twice to remove the traces of contaminants. DNA was eluted with Tris buffer, pH 8.5; its concentration and purity were measured using NanoDrop spectrophotometer. DNA was stored at −20 °C for further analysis.

### Methylation-specific polymerase chain reaction (MSP)

Using MSP assay, the methylation status of *ERCC1*, *hMLH1*, *ATM*, *CDKN2B* (*p15INK4B*), *p14ARF*, *CDKN2A* (*p16INK4A*), *RASSF1A*, *RUNX3*, *GATA6*, *NDRG2*, *PTEN*, and *RARβ* was assessed. Bisulfite modification of 500 ng DNA, preceding MSP, was performed using the EZ DNA methylation kit [Zymo Research Corp., USA, D5002], following manufacturer’s protocol. Commercially available completely methylated control DNA [CpG Methylated HeLa Genomic DNA, New England Biolabs], completely unmethylated control DNA [EpiTect Control DNA, unmethylated, Qiagen], and water served as positive, negative, and blank controls of MSP assay, respectively. DNA samples derived from the white blood cells from healthy volunteers as well as a brain fragment from a patient with brain hematoma served as normal control samples for DNA methylation analysis.

MSP assay was carried out in MyCycler Thermal Cycler with Gradient [Bio-Rad] or T100 [Bio-Rad]. Primer sequences for all the analyzed genes with their corresponding annealing temperature are presented in Table 1. PCR reactions were carried out using either FastStart Taq DNA polymerase [Roche Diagnostics], TrueStart Hot Start Taq DNA polymerase [Fermentas], Perpetual Taq DNA polymerase [EurX], or My Taq HS DNA polymerase [Bioline].

**Table 1:** Primer sequences used for MSP analysis, with PCR product size and primer annealing temperature

Gene symbol	Primer	Primer sequence (5′–3′)	Product size (bp)	Primer annealing temperature (°C)
*ERCC1*	MF	CGCGTTATCGCGGTTAAGT	223	60
	MR	ACCTTCCCCTCCTCTCAACTT		
	UF	TGGGTTGTGTGTTATTGTGGTTA	230	60
	UR	ACCTTCCCCTCCTCTCAACTT		
*hMLH1*	MF	ACGTAGACGTTTTATTAGGGTCGC	124	60
	MR	CCTCATCGTAACTACCCGCG		
	UF	TTTTGATGTAGATGTTTTATTAGGGTTGT	118	60
	UR	ACCACCTCATCATAACTACCCACA		
*ATM*	MF	GGTATGTTTATGCGTATTTAGTATTACGC	121	60
	MR	AACGCTAAATCGCTAACCATTAATAA		
	UF	TGGTATGTTTATGTGTATTTAGTATTATGT	123	56
	UR	AAACACTAAATCACTAACCATTAATAAA		
*CDKN2A* (*p16INK4A*)	MF	TTATTAGAGGGTGGGGCGGATCGC	150	65
	MR	GACCCCGAACCGCGACCGTAA		
	UF	TTATTAGAGGGTGGGGGTGGATTGT	151	60
	UR	CAACCCCAAACCACAACCATAA		
*p14ARF*	MF	GTGTTAAAGGGCGGCGTAGC	122	60
	MR	AAAACCCTCACTCGCGACGA		
	UF	TTTTTGGTGTTAAAGGGTGGTGTAGT	132	58
	UR	CACAAAAACCCTCACTCACAACAA		
*CDKN2B* (*p15INK4B*)	MF	GCGTTCGTATTTTGCGGTT	148	60
	MR	CGTACAATAACCGAACGACCGA		
	UF	TGTGATGTGTTTGTATTTTGTGGTT	154	60
	UR	CCATACAATAACCAAACAACCAA		
*RASSF1A*	MF	GTGTTAACGCGTTGCGTATC	94	60
	MR	AACCCCGCGAACTAAAAACGA		
	UF	TTTGGTTGGAGTGTGTTAATGTG	108	60
	UR	CAAACCCCACAAACTAAAAACAA		
*RUNX3*	MF	TTACGAGGGGCGGTCGTACGCGGG	220	66
	MR	AAAACGACCGACGCGAACGCCTCC		
	UF	TTATGAGGGGTGGTTGTATGTGGG	220	63
	UR	AAAACAACCAACACAAACACCTCC		
*GATA6*	MF	CGGGGTAGATTTCGGATTCGC	116	60
	MR	CAACCGAACCTCGAACGAACG		
	UF	GTGTGGGGTAGATTTTGGATTTGT	122	60
	UR	AAACAACCAAACCTCAAACAAACA		
*NDRG2*	MF	AGAGGTATTAGGATTTTGGGTACG	123	55
	MR	GCTAAAAAAACGAAAATCTCGC		
	UF	AGAGGTATTAGGATTTTGGGTATGA	125	55
	UR	CCACTAAAAAAACAAAAATCTCACC		
*PTEN*	MF	TTCGTTCGTCGTCGTCGTATTT	207	62
	MR	GCCGCTTAACTCTAAACCGCAA		
	UF	GTGTTGGTGGAGGTAGTTGTTT	162	62
	UR	ACCACTTAACTCTAAACCACAACCA		
*RARβ*	MF	TGTCGAGAACGCGAGCGATTC	148	60
	MR	CGACCAATCCAACCGAAACGA		
	UF	TTGGGATGTTGAGAATGTGAGTGATTT	154	60
	UR	CCTACTCAACCAATCCAACCAAAACAA		

The reaction conditions differed for each type of polymerase used, but generally after an initial step of heat denaturation at 95 °C for 1–4 min. (depending on the type of polymerase used), 35–38 cycles were carried out (95 °C for 15–30 s, appropriate annealing temperature for 30 s and 72 °C for 10–45 s), which were followed by final elongation at 72 °C for 5–7 min. The PCR products were separated by electrophoresis in 2 % agarose gel stained with ethidium bromide and visualized under UV illumination.

### Pyrosequencing (PSQ)

**Fig. 1:**
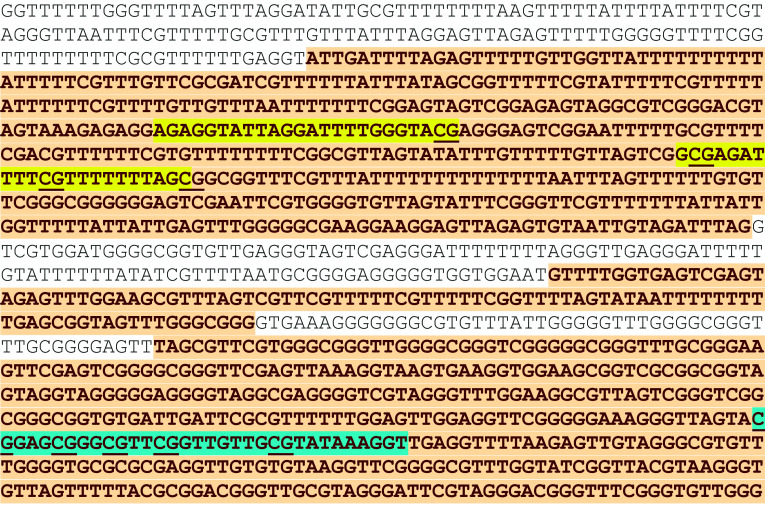
The fragment of *NDRG2* promoter (sequence after the bisulfite treatment), with the sequence analyzed using PSQ (highlighted with *blue*) and MSP (highlighted with *yellow*—primers MF and MR). Analyzed CpG dinucleotides are *underlined*

Pyrosequencing (PSQ) of *NDRG2* was performed on a PyroMark Vacuum Prep Workstation and Pyrosequencer PSQ™ 96 ID system, using PyroQ CpG™ software 1.0.9 (Biotage, Uppsala, Sweden), for quantification of the methylation level. Bisulfite-treated DNA samples were first amplified using FastStart Taq DNA Polymerase [Roche Diagnostics], with the forward primer: GGGCGGTGTGATTGATTC, and reverse, biotinylated primer: biotin-CCCACAATCTTCTCCCGTTCT (primers were designed using PyroQ CpG™ software 1.0.9). The 25 μl of reaction mixture contained 5 mM MgCl_2_, 10 × buffer, 0.2 mM dNTPs, 5 U/μl polymerase, 0.4 μM forward primer, 0.2 μl reverse primer, and water. The reaction conditions were as follows: 95 °C 4 min., 45 cycles of 95 °C for 30 s, 58 °C for 30 s, 72 °C for 45 s, followed by 72 °C for 7 min. The PCR product had 327 bp, and 2 μl of the product was visualized after ethidium bromide staining on 2 % agarose gel; the rest of the PCR product underwent PSQ. The analyzed region is presented in Fig. 1. The sequencing primer was as follows: GGGGGAAAGGGTTAGTA. The analyzed fragment spanned 5 CpG dinucleotides: T/CGGAGT/CGGGT/CGTTT/CGGTTGTTGT/CGTATAAAGGT, and the dispensation order was as follows: GTCGATGTCAGTCTGTCGTGTAGTCGTA.

As controls, completely methylated control DNA (CpG Methylated HeLa Genomic DNA, New England Biolabs), and completely unmethylated control DNA (EpiTect Control DNA, unmethylated, Qiagen), as well as DNA from normal, non-cancerous brain tissue were included in the assay. The reaction without any template DNA (blank) was also performed. Internal control for completion of the bisulfite treatment was included in the sequence. The cutoff value to distinguish between methylated and unmethylated samples was established at 11 %, based on the results obtained on control non-cancerous brain tissue, according to the previously described formula (Majchrzak-Celińska et al. 2015; Håvik et al. 2012).

### Statistical analysis

Css Statistica, version 10, was used for statistical analysis. Fisher’s exact test was used to determine the correlations between methylation frequency and WHO grade of the tumors (Stat Exact program). Spearman’s rank correlation coefficient was used to measure the dependence between MI and WHO grade of the tumor, as well as patient’s age. Student’s *t* test was applied to determine the relevance of Pearson’s R coefficient. Chi-squared test and Pearson’s R coefficient were used to determine the correlation between methylation analysis results and patient’s age. Kruskal–Wallis test was used to determine the dependence between histological type of the tumor and MI, differences in methylation levels determined by PSQ between tumors of different WHO grade and the dependences between methylation levels, when analyzed with PSQ and patient’s age. Mann–Whitney *U* test was used to determine the statistical significance of methylation levels in each CpG site in terms of patient’s sex.

## Results

### MSP analysis

**Fig. 2:**
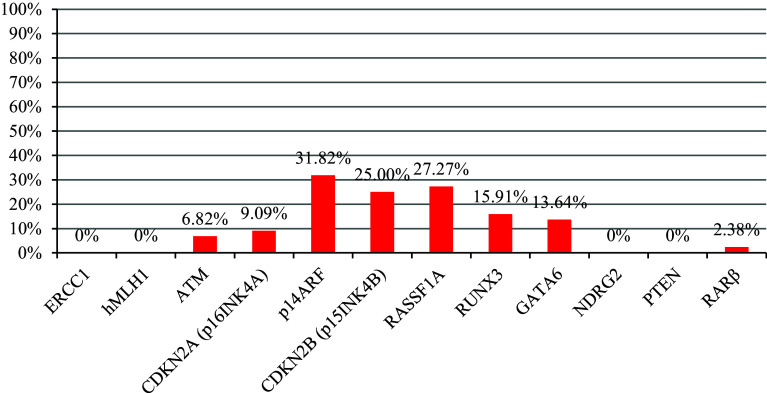
The frequency of gene promoter methylation in all samples analyzed

MSP analysis was applied to analyze the methylation status of 12 genes in 44 meningioma samples. The frequency of gene promoter methylation is presented in Fig. 2. The most frequently methylated gene was *p14ARF*—methylated in 31.82 % (14/44) of tumor samples, followed by *RASSF1A* methylated in 27.27 % (12/44) and *CDKN2B* (*p15INK4B*) which was methylated in 25.00 % (11/44) of cases. *RUNX3* and *GATA6* were found methylated in 15.91 % (7/44) and 13.64 % (6/44), respectively. Infrequent methylation was found for *CDKN2A* (*p16INK4A*) (9.09 %, 4/44), *ATM* (6.82 %, 3/44), and *RARβ* (2.38 %, 1/42) promoters. *ERCC1*, *hMLH1*, *NDRG2*, and *PTEN* were unmethylated in all samples analyzed (0 %, 0/44). The data illustrating the correlation of gene promoter methylation with WHO tumor grade are presented in Table 2. The median number of methylated genes was 1, and the highest MI was 0.33, indicating four methylated genes. *RUNX3* methylation was statistically correlated with higher grade meningiomas (*p* = 0.03), and *RASSF1A* was more frequently methylated in men than women (*p* = 0.04).

**Table 2:** Correlation of gene promoter methylation with WHO tumor grade

Gene	Methylation status	WHO grade II	WHO grade I/II	WHO grade I	Fisher’s exact test
*ERCC1*	M	0	0	0	–
	U	9	2	33	
*hMLH1*	M	0	0	0	–
	U	9	2	33	
*ATM*	M	0	0	3	*p* = 0.99
	U	9	2	30	
*CDKN2A* (*p16INK4A*)	M	1	0	3	*p* = 0.74
	U	8	2	30	
*p14ARF*	M	4	1	9	*p* = 0.38
	U	5	1	24	
*CDKN2B* (*p15INK4B*)	M	3	0	8	*p* = 0.82
	U	6	2	25	
*RASSF1A*	M	5	0	7	*p* = 0.15
	U	4	2	26	
*RUNX3*	M	5	0	5	*p* = 0.03*
	U	4	2	28	
*GATA 6*	M	0	0	6	*p* = 0.49
	U	9	2	27	
*NDRG2*	M	0	0	0	–
	U	9	2	33	
*PTEN*	M	0	0	0	–
	U	9	2	33	
*RARβ*	M	1	0	0	*p* = 0.2
	U	6	2	33	

* Significant value

### *NDRG2* pyrosequencing (PSQ)

**Fig. 3:**
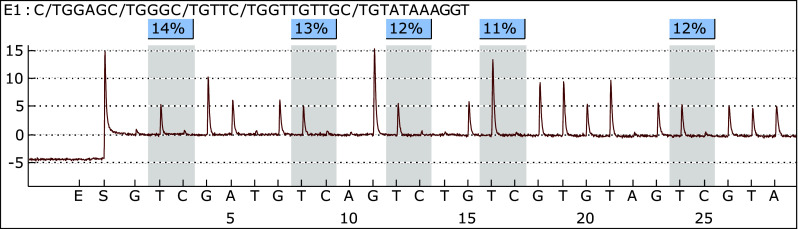
Representative pyrogram—sample T1—fibrous meningioma (WHO grade I)

**Fig. 4:**
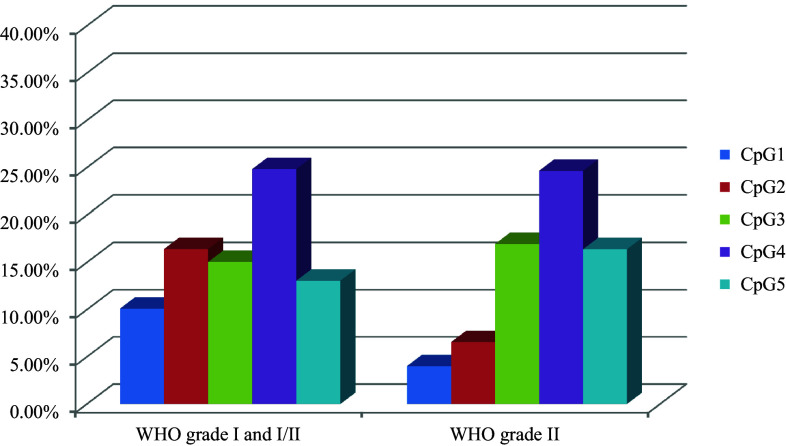
The relationship between average *NDRG2* methylation level in each CpG dinucleotide and WHO grades of analyzed meningiomas. Each CpG dinucleotide is represented by *different color*

Twenty-five meningioma samples were analyzed using PSQ. Sixty-eight percent of the samples (17/25) represented benign tumors (WHO grade I); however, one sample was classified as I/II grade, and seven were classified as grade II. Representative pyrogram is shown in Fig. 3. Totally, 84.00 % (21/25) of tumors showed a mean methylation signal above the level represented by normal, non-cancerous brain tissue. The highest methylation level was found for CpG4, whereas the lowest for CpG1. The average methylation of the five CpG sites analyzed in the methylated group were as follows: CpG1—8.17 %, CpG2—14.05 %, CpG3—17.14 %, CpG4—27.60 %, and CpG5—14.09 %. The mean methylation levels for tumor WHO grade I and I/II were 15.90 % (±5.53 %) and for grade II 13.71 % (±8.41 %) and thus could not differentiate between tumor WHO grades. The relationship between average *NDRG2* methylation level in each CpG dinucleotide and WHO grades of analyzed meningiomas is presented in Fig. 4. Kruskal–Wallis test revealed that the average methylation level of all five CpG sites correlates with patient’s age—higher methylation levels were observed in older patients, as compared with younger ones (*p* = 0.03). According to Mann–Whitney’s *U* test, women had statistically higher methylation levels in CpG2 as compared to men (*p* = 0.01).

The comparison of *NDRG2* promoter methylation profile obtained by MSP and PSQ revealed that 4 out of 25 samples gave concordant results when analyzed using MSP and PSQ, with the cutoff value determined to be 11 %. The high discordant rate was expected since two different fragments of the promoter were analyzed (the distance between them was 643 nt).

## Discussion

Recent genome-scale DNA methylation analyses of different subtypes of meningiomas although provided novel insights into the molecular pathophysiology of malignant transformation did not indicate the definite markers of the development of these tumors (Kishida et al. 2012; Gao et al. 2013). Therefore, a crucial methylation marker to predict a greater malignant potential of meningiomas still remains to be established.

In our study, DNA methylation of a panel of genes was assessed in a group of patients with benign or atypical meningiomas. The panel included genes encoding DNA repair proteins (*ERCC1*, *hMLH1*, and *ATM*), cell cycle regulators (genes encoded in INK4B-ARF-INK4A locus, *RASSF1A*, and *PTEN*), and genes involved in cell differentiation and proliferation (*RARβ*, *GATA6*, *NDRG2*, and *RUNX3*). Our earlier study revealed that MI of these genes, i.e., the number of methylated genes divided by the number of all genes analyzed in the panel (Chen et al. 2011) correlated with glioma aggressiveness (Majchrzak-Celińska et al. 2015). The results of this study showed that such correlation does not occur in meningiomas. Although tumors of the same grade according to WHO criteria had differently methylated genes, the number of methylated genes for each individual patient was low, with the median of one and the maximum of four methylated genes out of 12. However, the analysis of individual genes methylation provided some interesting observations. The most important finding of our current study was the methylation of *RUNX3*, which was found in more than half of atypical meningiomas [55.56 %] and also in a subset of benign meningiomas [16.67 %]. More importantly, according to Fisher’s exact test, *RUNX3* methylation correlated with the presence of more aggressive, atypical meningiomas (*p* = 0.03).

The Runt-related transcription factors (RUNX) are involved in developmental processes and, through multiple-interacting proteins, have been implicated in diverse signaling pathways and cellular processes (Chuang et al. 2013). The frequent inactivation of *RUNX* genes in cancer indicates crucial roles for RUNX in tumor suppression; however, they have also been characterized as oncogenes, depending on the context (Blyth et al. 2005). The role of *RUNX3* methylation has been intensively studied in gastric cancers (Fan et al. 2011); however, data relating CNS tumors are very limited and apply only to small groups of patients  (Avci et al. 2014). Thus, although the results of our present study require confirmation on larger patient cohorts, they suggest that *RUNX3* methylation might be considered as the promising candidate biomarker for the assessment of meningioma aggressivenes.

Recently, significant differences in the expression of *NDRG2* gene were reported between primary and recurrent meningioma groups, and benign and atypical meningiomas (Skiriute et al. 2011). Earlier, the downregulation of this gene was described in a subset of lower-grade meningiomas, including atypical meningiomas with clinically aggressive behavior, which was associated with hypermethylation of the *NDRG2* promoter (Lusis et al. 2005). In this study, we analyzed *NDRG2* promoter methylation using PSQ technique, because the sequence, reported to be a target of aberrant methylation in colon cancer (Piepoli et al. 2009) and used in our MSP analysis, was found unmethylated in all meningioma samples analyzed. The analysis of five CpG sites in *NDRG2* promoter revealed methylation in a large subset of meningiomas. Eighty-four percent of tumor samples showed a mean methylation signal above the level found in normal, non-cancerous brain tissue. However, the mean methylation levels of five CpGs were below 20 % for all meningioma grades and whether this mild methylation has any significant impact on mRNA levels remains to be determined. However, higher methylation levels in CpG2 were observed in women than men, and the average methylation level of all five CpG sites correlated with patient’s age. Thus, the results of our study confirm that mild *NDRG2* methylation is a common event in meningioma formation, but clinical application of this observation seems to be limited. Moreover, PSQ analysis of a sequence within 5′UTR was proven to be a reliable method for its analysis.

Methylation of *RASSF1A* promoter is considered as an early and frequent event in tumorigenesis, and there are attempts to apply *RASSF1A* methylation as a diagnostic marker in cancer screening (Richter et al. 2009). The results of our study revealed that *RASSF1A* is methylated in 27.27 % of meningioma samples, and the frequency of gene promoter methylation is increasing with the tumor grade (25.00 % of tumors grade I and I/II, and 55.56 % of tumors grade II). Thus, we confirm the earlier findings of Nakane et al. (2007), which showed *RASSF1A* promoter methylation in 18.2, 63.6, and 42.9 % of the grade I, II, and III of meninigiomas, respectively. Overall, these results suggest that *RASSF1A* methylation is likely to play an important role in both meningioma development and progression. However, because this gene is frequently methylated in other malignancies (Richter et al. 2009), its role as meningioma biomarker is limited and should be considered only in a panel of other frequently methylated genes.

The panel of genes whose promoter methylation was analyzed in this study included also other commonly hypermethylated ones, such as those located in the INK4B-ARF-INK4A locus. Inactivation of the G1/S-phase cell cycle checkpoint was reported to be an important aberration in anaplastic meningiomas (Boström et al. 2001), and it was suggested that deregulations of p14-MDM2-p53 pathway may contribute to the malignant progression of those tumors (Amatya et al. 2004). The results of our study pointed to *p14ARF* gene as the most frequently methylated one in INK4B-ARF-INK4A locus (31.82 % of all samples), followed by p*15INK4B* (*CDKN2B*) (25.00 %) and *p16INK4A* (*CDKN2A*) (9.09 %). Methylation of these genes characterized both benign as well as atypical meningiomas. These findings indicate that INK4B-ARF-INK4A locus is a target of aberrant methylation in meningiomas and that at least in some of those tumors the loss of cell cycle control is due to epigenetic silencing of *p14ARF*, *p15INK4B*, and/or *p16INK4A* genes. According to the two-hit model of Knudson, DNA methylation can be a second hit inactivating those tumor suppressors, following genetic mutations which are also often present in meningiomas in this locus (Boström et al. 2001; Knudson 1996). Similar findings concerning DNA methylation in meningiomas were recently published by Aydemir et al. (2012), who found *p16INK4A* to be methylated in 8.3 % [3/36] of cases and Liu et al. (2005) reporting methylation of this gene in 10 % of samples. Amatya et al. (2004) found methylation of *p14ARF* gene in 5 of 58 cases of benign meningiomas (8.6 %), 2 of 10 cases of atypical meningiomas (20 %), and 2 of 4 cases of anaplastic meningiomas (50 %). However, the utility of INK4B-ARF-INK4A locus methylation in terms of clinically useful biomarker seems limited. Linsler et al. (2014) reported that methylation of *p16INK4A* is not associated with recurrence, higher grade, or chromosomal aberrations observed in meningiomas. What is more neither age nor sex had significant influence on tumor recurrence, with regard to the *p16INK4A* methylation status (Linsler et al. 2014).

Recently, *GATA6* gene was found to be frequently methylated in glioma patients (Skiriute et al. 2012; Cecener et al. 2012; Martinez et al. 2009). Moreover, significant association of this gene methylation with unfavorable patient survival was found (Martinez et al. 2009). In meningiomas, to our best knowledge, the methylation of this gene has not been analyzed. Our results indicate that similarly to gliomas, *GATA6* promoter is also methylated in meningiomas, however, with significantly lower frequency (13.64 vs 30–68 % in glioma). Thus, *GATA6* promoter methylation in meningioma may play certain role in menigioma development, although cannot be considered as a biomarker candidate.

The other genes analyzed in this study, namely *ATM* and *RARβ*, were found to be methylated in a marginal number of patients (*ATM*—6.82 %, *RARβ*—2.38 %), while *ERCC1*, *hMLH1*, and *PTEN* were not methylated at all. The low frequency or lack of methylation of those genes suggests that their promoter methylation does not play a major role in the development of meningioma.

In summary, this study provided evidence that *p14ARF*, *RASSF1A*, *p15INK4B *(*CDKN2B*), *RUNX3*, *GATA6*, *p16INK4A *(*CDKN2A*), *NDRG2* and to lesser extent *ATM* and *RARβ* promoter methylation are associated with the development of meningiomas. *RUNX3* methylation correlates with meningioma WHO grade and, therefore, can be used as a potential indicator of tumor aggressiveness. Despite the methylation of *NDRG2* in the majority of meningiomas, it cannot be considered as a diagnostic biomarker because its methylation level is only slightly elevated in comparison to non-cancerous brain tissue. Overall, the results of this study confirm that DNA methylation plays an important role in the pathogenesis of meningiomas. Further investigations, particularly concerning *RUNX3* methylation, are required in order to assess clinically useful potential of the genes analyzed in this study.
